# A new monoclinic polymorph of 1,1′-bis­(di­phenyl­thio­phosphor­yl)ferrocene

**DOI:** 10.1107/S2056989015012682

**Published:** 2015-07-04

**Authors:** Yee Seng Tan, Chien Ing Yeo, Edward R. T. Tiekink

**Affiliations:** a Department of Chemistry, University of Malaya, 50603 Kuala Lumpur, Malaysia

**Keywords:** crystals structure, ferrocene derivative, polymorph, conformation

## Abstract

The title compound, [Fe(C_17_H_14_PS)_2_], is a second monoclinic polymorph (*P*2_1_/*c*, with *Z*′ = 1) of the previously reported monoclinic (*C*2/*c*, with *Z*′ = 1/2) form [Fang *et al.* (1995 ▸). *Polyhedron*, **14**, 2403–2409]. In the new form, the S atoms lie to the same side of the mol­ecule with the pseudo S—P⋯P—S torsion angle being −53.09 (3)°. By contrast to this almost *syn* disposition, in the *C*2/*c* polymorph, the Fe atom lies on a centre of inversion so that the S atoms are strictly *anti*, with a pseudo-S—P⋯P—S torsion angle of 180°. The significant difference in mol­ecular conformation between the two forms does not result in major perturbations in the P=S bond lengths nor in the distorted tetra­hedral geometries about the P atoms. The crystal packing of the new monoclinic polymorph features weak Cp—C—H⋯π(phen­yl) inter­actions consolidating linear supra­molecular chains along the *a* axis. These pack with no directional inter­actions between them.

## Abstract 2

In 1,1′-bis­(di­phenyl­thio­phosphor­yl)ferrocene, the S atoms lie to the same side of the mol­ecule. By contrast to this almost *syn* disposition, in a previous form, the Fe atom lies on a centre of inversion so that the S atoms are strictly *anti*.

## Chemical context   

Phosphanegold(I) di­thio­carbamates, *R*_3_PAu(S_2_CN*R*′_2_), attract on-going inter­est owing to impressive biological activities against both cancer (Jamaludin *et al.*, 2013 ▸) and microbes (Sim *et al.*, 2014 ▸). It was in the course of these studies that crystals of the title compound, dppfS_2_, an oxidation product of 1,1′-bis­(di­phenyl­phosphane)ferrocene (dppf), were isolated as orange needles, being a side-product of a reaction, see *Synthesis and crystallization* for details. Crystallography shows the title compound to be a new monoclinic polymorph of a previously described *C*2/*c* form (Fang *et al.*, 1995 ▸). Herein, details of the new polymorph are described along with a comparison with the original polymorph. A discussion of the key structural characteristics of related dppf*Y*_2_, *Y* = 0, O, S and Se, structures ensues.
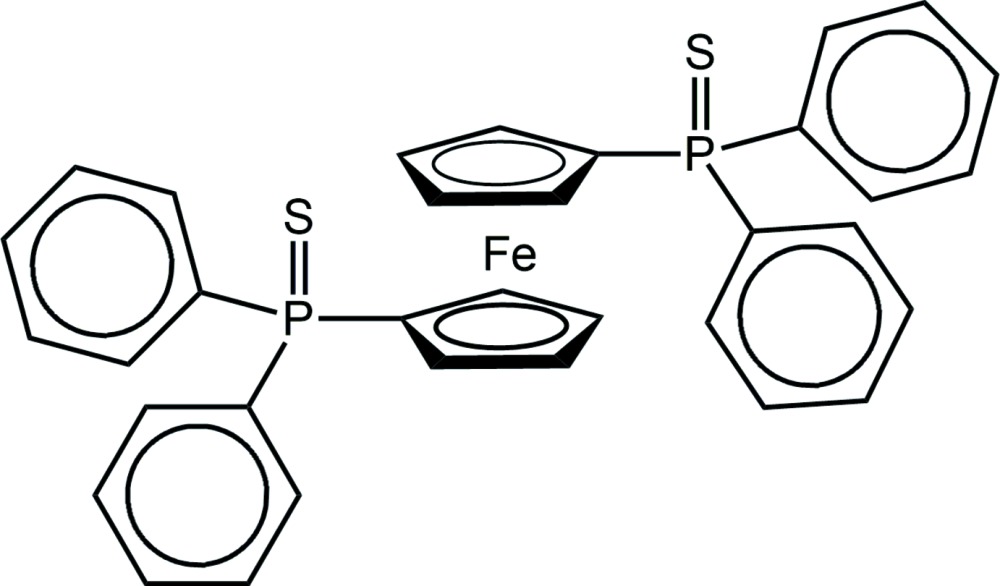


## Structural commentary   

**Figure 1:**
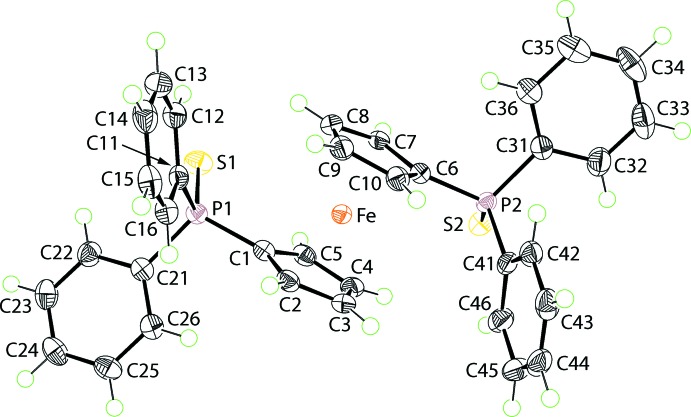
The mol­ecular structure of the new *P*2_1_/*c* polymorph of dppfS_2_, showing the atom-labelling scheme and displacement ellipsoids at the 50% probability level.

The mol­ecular structure of dppfS_2_ is shown in Fig. 1 ▸ and comprises two Ph_2_P=S units linked *via* the P atoms through a C_5_H_4_FeC_5_H_4_ link. The S atoms lie to the same side of the mol­ecule and might be described as having a *syn* conformation. When viewed down the P⋯P axis, the S atoms are gauche with the pseudo S—P⋯P—S torsion angle being −53.09 (3)°. This represents the major difference between dppfS_2_ and its *C*2/*c*–dppfS_2_ polymorph (Fang *et al.*, 1995 ▸). In the latter the Fe atom lies on a crystallographic centre of inversion, implying the S atoms are *anti* and that the pseudo S—P⋯P—S torsion angle is 180°.

**Figure 2:**
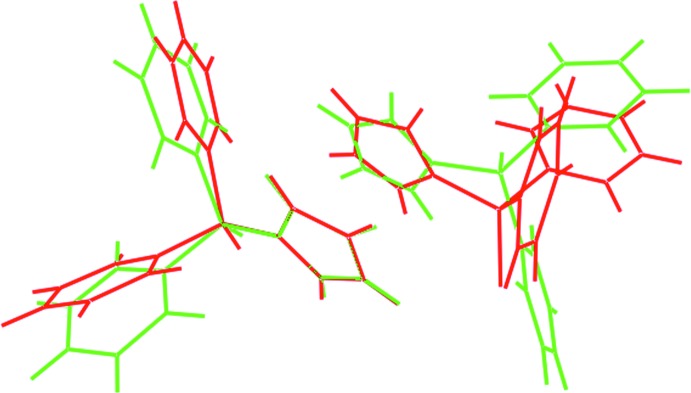
Overlay diagram of the *P*2_1_/*c* (red image) and *C*2/*c* (green) polymorphs overlapped so that one Cp ring of each mol­ecule is coincident.

The conformational differences in the polymorphs are highlighted in the overlay diagram shown in Fig. 2 ▸. The Fe atom is equally disposed from the centroids of the very nearly eclipsed Cp rings: Fe⋯*Cg*(C1–C5) and *Cg*(C6–C10) are 1.6487 (8) and 1.6451 (8) Å, respectively, and the *Cg*(C1–C5)⋯Fe⋯*Cg*(C6–C10) angle is 178.92 (5)°. The comparable parameters for the *C*2/*c*–dppfS_2_ polymorph are 1.650 (3) Å and 180°, and the Cp rings are strictly staggered when viewed down the *Cg*(C1–C5)⋯Fe⋯*Cg*(C1–C5)^i^ axis. In dppfS_2_, the P=S bond lengths are experimentally distinct, *i.e*. P1=S1 of 1.9449 (6) Å is shorter than P2=S2 of 1.9530 (6) Å, with the former being equivalent to P1=S1 of 1.9384 (18) Å in *C*2/*c*–dppfS_2_. Finally, the P1 and P2 atoms have distorted tetra­hedral environments with the range of angles subtended at P1 of 103.94 (7)–113.78 (6)° being comparable to those subtended at P2, *i.e*. 105.55 (7)–114.92 (5)°; the equivalent range of angles in *C*2/*c*–dppfS_2_ is 104.8 (2)–114.28 (15)°. In each case, the angles involving the S atom are wider than those involving C atoms only, and the narrowest angle always involves the two *ipso*-C atoms.

## Supra­molecular features   

**Figure 3:**
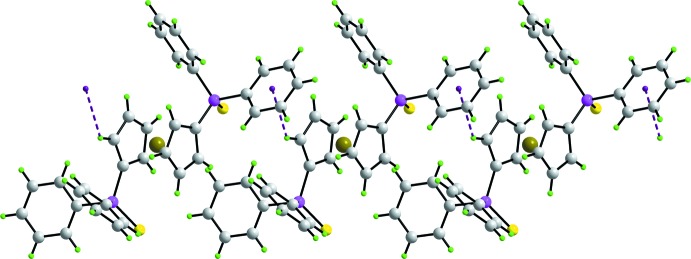
Supra­molecular chain along the *a* axis sustained by C—H⋯π inter­actions shown as purple dashed lines.

**Figure 4:**
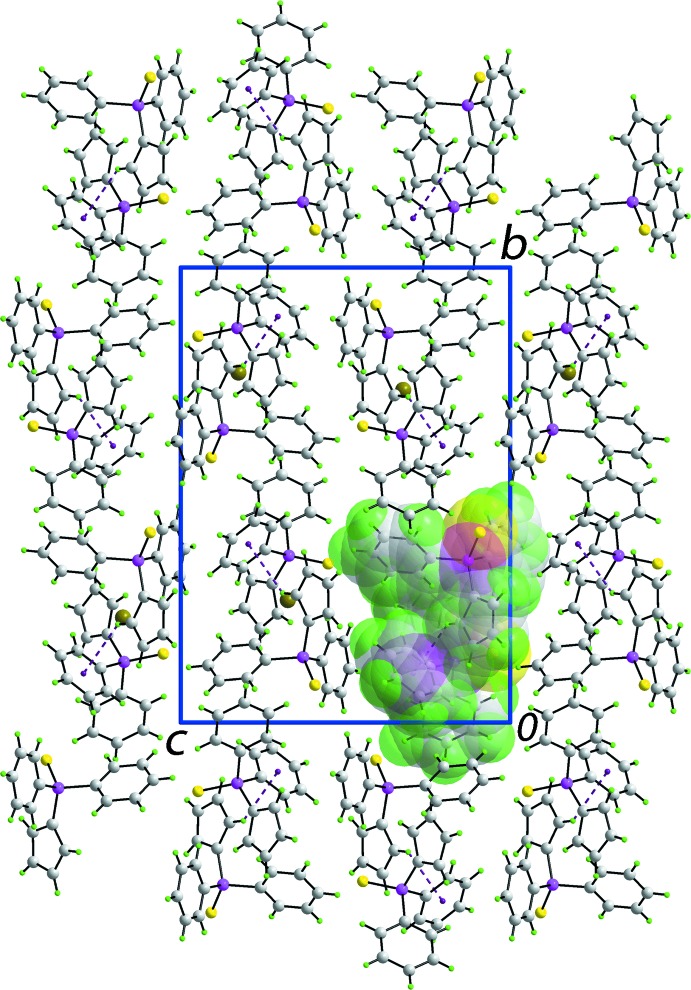
Unit-cell contents shown in projection down the *a* axis. The C—H⋯π contacts are shown purple dashed lines. One of the supra­molecular chains shown in Fig. 3 ▸ has been highlighted in space-filling mode.

Globally, the crystal packing features columns of mol­ecules aligned along the *a* axis. Based on the distance criteria employed in *PLATON* (Spek, 2009 ▸), the most notable inter­molecular contact operating in the crystal structure is a Cp-C2—H2⋯π(C31–C36) inter­action, Table 1 ▸, that connects translationally related mol­ecules into a supra­molecular chain along the *a* axis, Fig. 3 ▸. Chains pack with no specific directional inter­actions between them, Fig. 4 ▸. In the *C*2/*c*–dppfS_2_ polymorph, the most prominent directional inter­action is a weak C—H⋯S contact. The crystal packing efficiencies calculated by *PLATON* (Spek, 2009 ▸) are 69.3 and 67.2%, respectively, indicating the more symmetric structure packs less efficiently.

## Database survey   

Subsequent to the report of the *C*2/*c* form by Fang *et al.* (1995 ▸), a second report appeared (Pilloni *et al.*, 1997 ▸). In the latter analysis, the authors suggested that *Cc* was the correct space group. The assignment of *C*2/*c* was later confirmed as being correct (Clemente & Marzotto, 2004 ▸).

The structures of several oxidation products of dppf, Ph_2_P(=*Y*)C_5_H_4_FeC_5_H_4_P(=*Y*)Ph_2_, *Y* = 0, O, S and Se, have been described in the crystallographic literature. The parent compound, *i.e*. with *Y* = lone pair, has the Fe atom situated on a centre of inversion (Casellato *et al.*, 1988 ▸). When *Y* = O, an unsolvated form has been reported with the Fe atom again located on a centre of inversion (Pilloni *et al.*, 1993 ▸). A monohydrate (Bar *et al.*, 2008 ▸; Bolte *et al.*, 1997 ▸) as well as a dihydrate (Munyejabo *et al.*, 1994 ▸; Fang *et al.*, 1995 ▸) have also been described. In the former, the O atoms are approximately *syn* while the latter is centrosymmetric, *i.e*. resembling the situation with the *Y* = S polymorphs. Finally, when *Y* = Se, centrosymmetric structures are found in the unsolvated form (Arsenyan *et al.*, 2012 ▸) as well as in the CH_2_Cl_2_ monosolvate (Pilloni *et al.*, 1997 ▸). Clearly, there is significant conformational flexibility in the Ph_2_P(=*Y*)C_5_H_4_FeC_5_H_4_P(=*Y*)Ph_2_, *Y* = 0, O, S and Se, compounds suggesting a low energy barrier for the inter­change from one conformation to another. The structural data for Ph_2_P(=*Y*)C_5_H_4_FeC_5_H_4_P(=*Y*)Ph_2_ are summarized in Table 2 ▸.

The dppfS_2_ mol­ecule can function as a ligand in metal complexes, often forming zero-dimensional mononuclear species (*e.g*. Gimeno *et al.*, 1995 ▸, 2000 ▸; Pilloni *et al.*, 1997 ▸) but sometimes binuclear species (Pilloni *et al.*, 1998 ▸). Two examples exist whereby dppfS_2_ bridges metal toms to form one-dimensional coordination polymers (Gimeno *et al.*, 1998 ▸, 2000 ▸).

## Synthesis and crystallization   

Two solutions were prepared. Firstly, a solution sodium salt of piperazine di­thio­carbamate (0.7 mmol) was prepared by dissolving piperazine (0.0582 g) in aceto­nitrile (50 ml). NaOH (112 µl of 50% *w*/*w*) and CS_2_ (84.6 µl) were added. Chloro­form (150 ml) was then added and the reaction mixture was stirred for 2 h. A second solution containing [1,1′-bis­(di­phenyl­phosphane)ferrocene]bis­[chlorido­gold(I)] (1.4 mmol) was prepared by dissolving potassium tetra­chlorido­aurate(III) (1.06 g) in a solvent mixture of acetone and water (1:2, 45 ml). Drop-wise addition of sodium sulfite (0.71 g) in water (10 ml) followed. Upon discolouration, bis­(di­phenyl­phos­phane)ferrocene (dppf, 0.78 g) in chloro­form (25 ml) was added. After stirring for 15 mins, the resulting gold precursor was extracted with chloro­form (150 ml). Aceto­nitrile (50 ml) was added to this to form solvent mixture of chloro­form and aceto­nitrile (3:1). The solution containing the di­thio­carbamate was added to that containing the gold precursor. The resulting mixture was stirred for 3 h. and then filtered. After three weeks, orange needles appeared, along with the precipitate, and these were subjected to the crystallographic study. Yield: 0.0890 g, 10.3% (based on dppf). M.p.: 519.5–519.9 K. IR: ν(P=S) 628 (*m*).

## Refinement   

Crystal data, data collection and structure refinement details are summarized in Table 3 ▸. Carbon-bound H-atoms were placed in calculated positions (C—H = 0.95 Å) and were included in the refinement in the riding-model approximation, with *U*_iso_(H) set to 1.2*U*_equiv_(C).

## Supplementary Material

Crystal structure: contains datablock(s) I, global. DOI: 10.1107/S2056989015012682/hg5450sup1.cif

Structure factors: contains datablock(s) I. DOI: 10.1107/S2056989015012682/hg5450Isup2.hkl

CCDC reference: 1409866

Additional supporting information:  crystallographic information; 3D view; checkCIF report

## Figures and Tables

**Table 1:** Hydrogen-bond geometry (, ) *Cg*1 is the centroid of the C31C36 benzene ring.

*D*H*A*	*D*H	H*A*	*D* *A*	*D*H*A*
C2H2*Cg*1^i^	0.95	2.92	3.6111(18)	130

Symmetry code: (i) 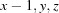.

**Table 2:** Summary of structural data () for Ph_2_P(*Y*)C_5_H_4_FeC_5_H_4_P(*Y*)Ph_2_

*Y*	Symmetry	*Y*PP*Y*	Solvent	CSD refcode^*a*^	Reference
O	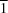	180		KADXAO	Casellato *et al.* (1988 ▸)
O	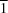	180		WARMUX	Pilloni *et al.* (1993 ▸)
O		155.57(18)	H_2_O	RUVJEX01	Bar *et al.* (2008 ▸)
O	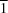	180	2H_2_O	HATTUR	Munyejabo *et al.* (1994 ▸)
S	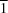	180		ZEQSOD	Fang *et al.* (1995 ▸)
S		53.09(3)			This work
Se	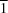	180		KIHWAB	Arsenyan *et al.* (2012 ▸)
Se	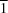	180	CH_2_Cl_2_	RIPTIT	Pilloni *et al.* (1997 ▸)

Note: (*a*) Cambridge Structural Database (Groom Allen, 2014 ▸), Version 5.35.

**Table 3:** Experimental details

Crystal data	
Chemical formula	[Fe(C_17_H_14_PS)_2_]
*M*_r_	618.47
Crystal system, space group	Monoclinic, *P*2_1_/*c*
Temperature (K)	100
*a*, *b*, *c* ()	8.7451(3), 21.2453(6), 15.4537(5)
()	95.631(3)
*V* (^3^)	2857.32(16)
*Z*	4
Radiation type	Mo *K*
(mm^1^)	0.81
Crystal size (mm)	0.25 0.25 0.25

Data collection	
Diffractometer	Agilent Technologies SuperNova Dual diffractometer with an Atlas detector
Absorption correction	Multi-scan (*CrysAlis PRO*; Agilent, 2013 ▸)
*T*_min_, *T*_max_	0.751, 1.000
No. of measured, independent and observed [*I* > 2(*I*)] reflections	31395, 6509, 5701
*R*_int_	0.036
(sin /)_max_ (^1^)	0.650

Refinement	
*R*[*F* ^2^ > 2(*F* ^2^)], *wR*(*F* ^2^), *S*	0.029, 0.073, 1.05
No. of reflections	6509
No. of parameters	352
H-atom treatment	H-atom parameters constrained
_max_, _min_ (e ^3^)	0.40, 0.24

Computer programs: *CrysAlis PRO* (Agilent, 2013 ▸), *SHELXS97* (Sheldrick, 2008 ▸), *SHELXL2014* (Sheldrick, 2015 ▸), *ORTEP-3 for Windows* (Farrugia, 2012 ▸), *QMol* (Gans Shalloway, 2001 ▸), *DIAMOND* (Brandenburg, 2006 ▸) and *publCIF* (Westrip, 2010 ▸).

## supplementary crystallographic information

### Crystal data

**Table:**

[Fe(C_17_H_14_PS)_2_]	*F*(000) = 1280
*M**_r_* = 618.47	*D*_x_ = 1.438 Mg m^−^^3^
Monoclinic, *P*2_1_/*c*	Mo *K*α radiation, λ = 0.71073 Å
*a* = 8.7451 (3) Å	Cell parameters from 11974 reflections
*b* = 21.2453 (6) Å	θ = 3.9–29.3°
*c* = 15.4537 (5) Å	µ = 0.81 mm^−^^1^
β = 95.631 (3)°	*T* = 100 K
*V* = 2857.32 (16) Å^3^	Prism, orange
*Z* = 4	0.25 × 0.25 × 0.25 mm

### Data collection

**Table:**

Agilent Technologies SuperNova Dual diffractometer with an Atlas detector	6509 independent reflections
Radiation source: SuperNova (Mo) X-ray Source	5701 reflections with *I* > 2σ(*I*)
Mirror monochromator	*R*_int_ = 0.036
Detector resolution: 10.4041 pixels mm^-1^	θ_max_ = 27.5°, θ_min_ = 2.8°
ω scan	*h* = −10→11
Absorption correction: multi-scan (*CrysAlis PRO*; Agilent, 2013)	*k* = −23→27
*T*_min_ = 0.751, *T*_max_ = 1.000	*l* = −19→20
31395 measured reflections	

### Refinement

**Table:**

Refinement on *F*^2^	0 restraints
Least-squares matrix: full	Hydrogen site location: inferred from neighbouring sites
*R*[*F*^2^ > 2σ(*F*^2^)] = 0.029	H-atom parameters constrained
*wR*(*F*^2^) = 0.073	*w* = 1/[σ^2^(*F*_o_^2^) + (0.0304*P*)^2^ + 1.6529*P*] where *P* = (*F*_o_^2^ + 2*F*_c_^2^)/3
*S* = 1.05	(Δ/σ)_max_ = 0.001
6509 reflections	Δρ_max_ = 0.40 e Å^−^^3^
352 parameters	Δρ_min_ = −0.24 e Å^−^^3^

### Special details

**Table:**

Geometry. All e.s.d.'s (except the e.s.d. in the dihedral angle between two l.s. planes) are estimated using the full covariance matrix. The cell e.s.d.'s are taken into account individually in the estimation of e.s.d.'s in distances, angles and torsion angles; correlations between e.s.d.'s in cell parameters are only used when they are defined by crystal symmetry. An approximate (isotropic) treatment of cell e.s.d.'s is used for estimating e.s.d.'s involving l.s. planes.

### Fractional atomic coordinates and isotropic or equivalent isotropic displacement parameters (Å^2^)

**Table:**

	*x*	*y*	*z*	*U*_iso_*/*U*_eq_	
Fe	0.34255 (2)	0.23085 (2)	0.17509 (2)	0.01395 (7)	
S1	0.24914 (5)	0.41754 (2)	0.09257 (3)	0.02424 (10)	
S2	0.69563 (5)	0.15111 (2)	0.05280 (3)	0.02111 (10)	
P1	0.10320 (5)	0.35615 (2)	0.12875 (3)	0.01579 (9)	
P2	0.65005 (5)	0.13323 (2)	0.17155 (3)	0.01496 (9)	
C1	0.16059 (18)	0.27653 (7)	0.11083 (10)	0.0166 (3)	
C2	0.11134 (18)	0.21919 (7)	0.14866 (11)	0.0182 (3)	
H2	0.0388	0.2156	0.1903	0.022*	
C3	0.19051 (18)	0.16874 (8)	0.11258 (11)	0.0210 (3)	
H3	0.1802	0.1254	0.1262	0.025*	
C4	0.28756 (19)	0.19377 (8)	0.05290 (10)	0.0203 (3)	
H4	0.3530	0.1702	0.0196	0.024*	
C5	0.27019 (18)	0.26013 (8)	0.05146 (10)	0.0179 (3)	
H5	0.3221	0.2887	0.0172	0.021*	
C6	0.54850 (18)	0.19445 (7)	0.22183 (10)	0.0156 (3)	
C7	0.56320 (18)	0.26062 (7)	0.20595 (10)	0.0165 (3)	
H7	0.6280	0.2795	0.1674	0.020*	
C8	0.46383 (19)	0.29294 (8)	0.25808 (11)	0.0200 (3)	
H8	0.4509	0.3373	0.2604	0.024*	
C9	0.38708 (19)	0.24804 (8)	0.30613 (10)	0.0202 (3)	
H9	0.3140	0.2571	0.3460	0.024*	
C10	0.43834 (18)	0.18708 (8)	0.28431 (10)	0.0185 (3)	
H10	0.4056	0.1483	0.3070	0.022*	
C11	0.07025 (18)	0.36206 (7)	0.24275 (10)	0.0172 (3)	
C12	0.1599 (2)	0.40242 (8)	0.29758 (11)	0.0219 (3)	
H12	0.2401	0.4260	0.2759	0.026*	
C13	0.1318 (2)	0.40811 (9)	0.38407 (12)	0.0266 (4)	
H13	0.1934	0.4355	0.4215	0.032*	
C14	0.0144 (2)	0.37407 (9)	0.41629 (11)	0.0264 (4)	
H14	−0.0051	0.3786	0.4754	0.032*	
C15	−0.0743 (2)	0.33340 (8)	0.36210 (11)	0.0237 (4)	
H15	−0.1535	0.3095	0.3843	0.028*	
C16	−0.04748 (19)	0.32759 (8)	0.27542 (11)	0.0204 (3)	
H16	−0.1093	0.3001	0.2382	0.025*	
C21	−0.08745 (18)	0.36448 (7)	0.07138 (10)	0.0176 (3)	
C22	−0.1447 (2)	0.42511 (8)	0.05516 (11)	0.0220 (3)	
H22	−0.0829	0.4607	0.0717	0.026*	
C23	−0.2922 (2)	0.43325 (8)	0.01480 (11)	0.0254 (4)	
H23	−0.3320	0.4745	0.0049	0.030*	
C24	−0.3816 (2)	0.38158 (9)	−0.01103 (11)	0.0234 (4)	
H24	−0.4826	0.3874	−0.0385	0.028*	
C25	−0.32411 (19)	0.32144 (8)	0.00299 (11)	0.0227 (3)	
H25	−0.3850	0.2860	−0.0158	0.027*	
C26	−0.17703 (19)	0.31268 (8)	0.04459 (10)	0.0196 (3)	
H26	−0.1380	0.2713	0.0547	0.023*	
C31	0.82272 (18)	0.11941 (7)	0.24346 (10)	0.0177 (3)	
C32	0.9158 (2)	0.06866 (8)	0.22515 (13)	0.0268 (4)	
H32	0.8863	0.0421	0.1769	0.032*	
C33	1.0508 (2)	0.05695 (9)	0.27696 (13)	0.0328 (4)	
H33	1.1126	0.0218	0.2650	0.039*	
C34	1.0959 (2)	0.09614 (10)	0.34592 (13)	0.0314 (4)	
H34	1.1889	0.0880	0.3812	0.038*	
C35	1.0063 (2)	0.14718 (9)	0.36387 (11)	0.0267 (4)	
H35	1.0390	0.1746	0.4106	0.032*	
C36	0.86822 (19)	0.15844 (8)	0.31345 (11)	0.0201 (3)	
H36	0.8051	0.1928	0.3269	0.024*	
C41	0.53591 (18)	0.06263 (7)	0.18021 (11)	0.0176 (3)	
C42	0.53090 (19)	0.03306 (8)	0.26044 (11)	0.0205 (3)	
H42	0.5890	0.0493	0.3107	0.025*	
C43	0.4402 (2)	−0.02033 (8)	0.26646 (12)	0.0254 (4)	
H43	0.4356	−0.0404	0.3211	0.031*	
C44	0.3569 (2)	−0.04412 (8)	0.19325 (13)	0.0279 (4)	
H44	0.2945	−0.0803	0.1979	0.033*	
C45	0.3636 (2)	−0.01573 (8)	0.11305 (13)	0.0285 (4)	
H45	0.3070	−0.0327	0.0628	0.034*	
C46	0.4534 (2)	0.03775 (8)	0.10640 (11)	0.0230 (4)	
H46	0.4584	0.0573	0.0515	0.028*	

### Atomic displacement parameters (Å^2^)

**Table:**

	*U*^11^	*U*^22^	*U*^33^	*U*^12^	*U*^13^	*U*^23^
Fe	0.01307 (12)	0.01394 (11)	0.01483 (12)	−0.00055 (8)	0.00125 (9)	−0.00082 (8)
S1	0.0231 (2)	0.0228 (2)	0.0270 (2)	−0.00807 (17)	0.00339 (18)	0.00380 (17)
S2	0.0243 (2)	0.0204 (2)	0.0197 (2)	−0.00123 (16)	0.00761 (17)	−0.00035 (15)
P1	0.0150 (2)	0.01549 (19)	0.0169 (2)	−0.00153 (15)	0.00182 (15)	0.00147 (15)
P2	0.0147 (2)	0.01333 (18)	0.0172 (2)	−0.00070 (15)	0.00332 (16)	−0.00081 (15)
C1	0.0142 (7)	0.0186 (7)	0.0168 (8)	−0.0006 (6)	0.0000 (6)	0.0003 (6)
C2	0.0128 (7)	0.0204 (8)	0.0213 (8)	−0.0018 (6)	0.0010 (6)	0.0006 (6)
C3	0.0172 (8)	0.0185 (8)	0.0262 (9)	−0.0025 (6)	−0.0038 (7)	−0.0035 (6)
C4	0.0187 (8)	0.0240 (8)	0.0175 (8)	0.0008 (7)	−0.0019 (6)	−0.0068 (6)
C5	0.0153 (8)	0.0237 (8)	0.0144 (7)	−0.0005 (6)	0.0004 (6)	0.0001 (6)
C6	0.0145 (7)	0.0158 (7)	0.0161 (7)	0.0008 (6)	0.0002 (6)	−0.0009 (6)
C7	0.0134 (7)	0.0159 (7)	0.0195 (8)	−0.0021 (6)	−0.0014 (6)	−0.0006 (6)
C8	0.0193 (8)	0.0171 (8)	0.0226 (8)	0.0010 (6)	−0.0031 (7)	−0.0045 (6)
C9	0.0204 (8)	0.0254 (8)	0.0144 (7)	0.0039 (7)	0.0002 (6)	−0.0033 (6)
C10	0.0189 (8)	0.0198 (8)	0.0167 (8)	0.0011 (6)	0.0010 (6)	0.0017 (6)
C11	0.0171 (8)	0.0169 (7)	0.0175 (8)	0.0045 (6)	0.0009 (6)	0.0013 (6)
C12	0.0216 (8)	0.0196 (8)	0.0241 (9)	0.0020 (6)	0.0004 (7)	0.0009 (7)
C13	0.0279 (9)	0.0282 (9)	0.0225 (9)	0.0042 (7)	−0.0042 (7)	−0.0047 (7)
C14	0.0282 (9)	0.0328 (10)	0.0181 (8)	0.0119 (8)	0.0015 (7)	0.0017 (7)
C15	0.0214 (8)	0.0275 (9)	0.0229 (9)	0.0069 (7)	0.0063 (7)	0.0063 (7)
C16	0.0188 (8)	0.0211 (8)	0.0215 (8)	0.0028 (6)	0.0021 (7)	0.0008 (6)
C21	0.0170 (8)	0.0201 (8)	0.0157 (7)	0.0000 (6)	0.0024 (6)	0.0026 (6)
C22	0.0245 (9)	0.0190 (8)	0.0223 (8)	0.0011 (7)	0.0016 (7)	0.0013 (6)
C23	0.0271 (9)	0.0235 (8)	0.0257 (9)	0.0075 (7)	0.0030 (7)	0.0059 (7)
C24	0.0182 (8)	0.0338 (9)	0.0182 (8)	0.0023 (7)	0.0012 (7)	0.0066 (7)
C25	0.0188 (8)	0.0270 (9)	0.0220 (8)	−0.0049 (7)	0.0016 (7)	0.0033 (7)
C26	0.0186 (8)	0.0196 (8)	0.0208 (8)	0.0000 (6)	0.0036 (7)	0.0040 (6)
C31	0.0148 (7)	0.0173 (7)	0.0215 (8)	−0.0014 (6)	0.0039 (6)	0.0040 (6)
C32	0.0230 (9)	0.0218 (8)	0.0360 (10)	0.0026 (7)	0.0050 (8)	0.0002 (7)
C33	0.0226 (9)	0.0322 (10)	0.0444 (12)	0.0097 (8)	0.0064 (8)	0.0096 (9)
C34	0.0148 (8)	0.0469 (12)	0.0324 (10)	0.0004 (8)	0.0018 (7)	0.0179 (9)
C35	0.0211 (9)	0.0397 (10)	0.0194 (8)	−0.0078 (8)	0.0023 (7)	0.0066 (7)
C36	0.0167 (8)	0.0247 (8)	0.0195 (8)	−0.0018 (6)	0.0049 (6)	0.0031 (6)
C41	0.0163 (8)	0.0131 (7)	0.0239 (8)	0.0005 (6)	0.0041 (6)	−0.0022 (6)
C42	0.0169 (8)	0.0178 (8)	0.0269 (9)	0.0005 (6)	0.0028 (7)	0.0016 (7)
C43	0.0202 (9)	0.0201 (8)	0.0367 (10)	0.0017 (7)	0.0063 (8)	0.0074 (7)
C44	0.0203 (9)	0.0150 (8)	0.0489 (11)	−0.0025 (7)	0.0062 (8)	−0.0010 (8)
C45	0.0250 (9)	0.0218 (9)	0.0384 (10)	−0.0036 (7)	0.0008 (8)	−0.0094 (8)
C46	0.0244 (9)	0.0199 (8)	0.0250 (9)	−0.0006 (7)	0.0045 (7)	−0.0049 (7)

### Geometric parameters (Å, º)

**Table:**

Fe—C6	2.0269 (15)	C13—C14	1.387 (3)
Fe—C10	2.0341 (16)	C13—H13	0.9500
Fe—C1	2.0372 (16)	C14—C15	1.387 (3)
Fe—C2	2.0383 (16)	C14—H14	0.9500
Fe—C7	2.0421 (15)	C15—C16	1.388 (2)
Fe—C3	2.0470 (16)	C15—H15	0.9500
Fe—C5	2.0495 (16)	C16—H16	0.9500
Fe—C9	2.0569 (16)	C21—C26	1.390 (2)
Fe—C4	2.0589 (16)	C21—C22	1.396 (2)
Fe—C8	2.0597 (16)	C22—C23	1.387 (2)
S1—P1	1.9449 (6)	C22—H22	0.9500
S2—P2	1.9530 (6)	C23—C24	1.383 (3)
P1—C1	1.7936 (16)	C23—H23	0.9500
P1—C11	1.8171 (16)	C24—C25	1.382 (2)
P1—C21	1.8184 (16)	C24—H24	0.9500
P2—C6	1.7943 (16)	C25—C26	1.393 (2)
P2—C31	1.8089 (16)	C25—H25	0.9500
P2—C41	1.8139 (16)	C26—H26	0.9500
C1—C5	1.433 (2)	C31—C36	1.390 (2)
C1—C2	1.436 (2)	C31—C32	1.397 (2)
C2—C3	1.419 (2)	C32—C33	1.382 (3)
C2—H2	0.9500	C32—H32	0.9500
C3—C4	1.417 (2)	C33—C34	1.379 (3)
C3—H3	0.9500	C33—H33	0.9500
C4—C5	1.418 (2)	C34—C35	1.382 (3)
C4—H4	0.9500	C34—H34	0.9500
C5—H5	0.9500	C35—C36	1.392 (2)
C6—C7	1.435 (2)	C35—H35	0.9500
C6—C10	1.438 (2)	C36—H36	0.9500
C7—C8	1.419 (2)	C41—C46	1.393 (2)
C7—H7	0.9500	C41—C42	1.394 (2)
C8—C9	1.418 (2)	C42—C43	1.392 (2)
C8—H8	0.9500	C42—H42	0.9500
C9—C10	1.422 (2)	C43—C44	1.380 (3)
C9—H9	0.9500	C43—H43	0.9500
C10—H10	0.9500	C44—C45	1.385 (3)
C11—C12	1.392 (2)	C44—H44	0.9500
C11—C16	1.398 (2)	C45—C46	1.391 (2)
C12—C13	1.388 (3)	C45—H45	0.9500
C12—H12	0.9500	C46—H46	0.9500
			
C6—Fe—C10	41.47 (6)	C6—C7—Fe	68.78 (9)
C6—Fe—C1	168.45 (6)	C8—C7—H7	126.1
C10—Fe—C1	149.39 (7)	C6—C7—H7	126.1
C6—Fe—C2	148.69 (6)	Fe—C7—H7	126.3
C10—Fe—C2	115.49 (7)	C9—C8—C7	108.65 (14)
C1—Fe—C2	41.25 (6)	C9—C8—Fe	69.75 (9)
C6—Fe—C7	41.30 (6)	C7—C8—Fe	69.10 (9)
C10—Fe—C7	69.21 (6)	C9—C8—H8	125.7
C1—Fe—C7	129.88 (6)	C7—C8—H8	125.7
C2—Fe—C7	168.66 (6)	Fe—C8—H8	127.1
C6—Fe—C3	115.82 (6)	C8—C9—C10	108.19 (14)
C10—Fe—C3	107.00 (7)	C8—C9—Fe	69.96 (9)
C1—Fe—C3	68.71 (6)	C10—C9—Fe	68.80 (9)
C2—Fe—C3	40.66 (6)	C8—C9—H9	125.9
C7—Fe—C3	149.88 (7)	C10—C9—H9	125.9
C6—Fe—C5	129.07 (6)	Fe—C9—H9	126.9
C10—Fe—C5	167.58 (6)	C9—C10—C6	107.92 (14)
C1—Fe—C5	41.06 (6)	C9—C10—Fe	70.53 (9)
C2—Fe—C5	68.94 (7)	C6—C10—Fe	69.00 (9)
C7—Fe—C5	108.78 (6)	C9—C10—H10	126.0
C3—Fe—C5	68.22 (7)	C6—C10—H10	126.0
C6—Fe—C9	68.97 (6)	Fe—C10—H10	126.0
C10—Fe—C9	40.67 (6)	C12—C11—C16	119.58 (15)
C1—Fe—C9	117.30 (7)	C12—C11—P1	119.96 (13)
C2—Fe—C9	107.89 (7)	C16—C11—P1	120.43 (12)
C7—Fe—C9	68.40 (7)	C13—C12—C11	119.85 (16)
C3—Fe—C9	129.09 (7)	C13—C12—H12	120.1
C5—Fe—C9	151.07 (7)	C11—C12—H12	120.1
C6—Fe—C4	107.48 (6)	C14—C13—C12	120.49 (17)
C10—Fe—C4	128.77 (7)	C14—C13—H13	119.8
C1—Fe—C4	68.55 (6)	C12—C13—H13	119.8
C2—Fe—C4	68.40 (7)	C15—C14—C13	119.86 (16)
C7—Fe—C4	117.58 (7)	C15—C14—H14	120.1
C3—Fe—C4	40.37 (7)	C13—C14—H14	120.1
C5—Fe—C4	40.38 (6)	C14—C15—C16	120.05 (17)
C9—Fe—C4	167.28 (7)	C14—C15—H15	120.0
C6—Fe—C8	68.73 (6)	C16—C15—H15	120.0
C10—Fe—C8	68.36 (7)	C15—C16—C11	120.15 (16)
C1—Fe—C8	109.16 (6)	C15—C16—H16	119.9
C2—Fe—C8	129.99 (7)	C11—C16—H16	119.9
C7—Fe—C8	40.47 (6)	C26—C21—C22	119.68 (15)
C3—Fe—C8	167.84 (7)	C26—C21—P1	122.07 (12)
C5—Fe—C8	118.62 (7)	C22—C21—P1	118.24 (12)
C9—Fe—C8	40.28 (7)	C23—C22—C21	119.82 (16)
C4—Fe—C8	151.13 (7)	C23—C22—H22	120.1
C1—P1—C11	106.75 (7)	C21—C22—H22	120.1
C1—P1—C21	105.88 (7)	C24—C23—C22	120.32 (16)
C11—P1—C21	103.94 (7)	C24—C23—H23	119.8
C1—P1—S1	112.74 (6)	C22—C23—H23	119.8
C11—P1—S1	113.78 (6)	C25—C24—C23	120.12 (16)
C21—P1—S1	113.00 (5)	C25—C24—H24	119.9
C6—P2—C31	105.69 (7)	C23—C24—H24	119.9
C6—P2—C41	105.55 (7)	C24—C25—C26	120.06 (16)
C31—P2—C41	104.63 (7)	C24—C25—H25	120.0
C6—P2—S2	114.92 (5)	C26—C25—H25	120.0
C31—P2—S2	111.95 (6)	C21—C26—C25	119.97 (15)
C41—P2—S2	113.24 (6)	C21—C26—H26	120.0
C5—C1—C2	107.49 (14)	C25—C26—H26	120.0
C5—C1—P1	122.88 (12)	C36—C31—C32	119.35 (15)
C2—C1—P1	129.63 (12)	C36—C31—P2	122.64 (12)
C5—C1—Fe	69.93 (9)	C32—C31—P2	117.97 (13)
C2—C1—Fe	69.42 (9)	C33—C32—C31	120.22 (18)
P1—C1—Fe	126.24 (8)	C33—C32—H32	119.9
C3—C2—C1	107.67 (14)	C31—C32—H32	119.9
C3—C2—Fe	70.00 (9)	C34—C33—C32	120.14 (18)
C1—C2—Fe	69.33 (9)	C34—C33—H33	119.9
C3—C2—H2	126.2	C32—C33—H33	119.9
C1—C2—H2	126.2	C33—C34—C35	120.27 (17)
Fe—C2—H2	126.1	C33—C34—H34	119.9
C4—C3—C2	108.58 (14)	C35—C34—H34	119.9
C4—C3—Fe	70.27 (9)	C34—C35—C36	120.01 (17)
C2—C3—Fe	69.34 (9)	C34—C35—H35	120.0
C4—C3—H3	125.7	C36—C35—H35	120.0
C2—C3—H3	125.7	C31—C36—C35	119.97 (16)
Fe—C3—H3	126.3	C31—C36—H36	120.0
C3—C4—C5	108.26 (14)	C35—C36—H36	120.0
C3—C4—Fe	69.36 (9)	C46—C41—C42	119.88 (15)
C5—C4—Fe	69.45 (9)	C46—C41—P2	119.87 (13)
C3—C4—H4	125.9	C42—C41—P2	120.25 (12)
C5—C4—H4	125.9	C43—C42—C41	119.62 (16)
Fe—C4—H4	126.9	C43—C42—H42	120.2
C4—C5—C1	108.00 (14)	C41—C42—H42	120.2
C4—C5—Fe	70.17 (9)	C44—C43—C42	120.12 (17)
C1—C5—Fe	69.01 (9)	C44—C43—H43	119.9
C4—C5—H5	126.0	C42—C43—H43	119.9
C1—C5—H5	126.0	C43—C44—C45	120.61 (16)
Fe—C5—H5	126.4	C43—C44—H44	119.7
C7—C6—C10	107.38 (14)	C45—C44—H44	119.7
C7—C6—P2	125.42 (12)	C44—C45—C46	119.71 (17)
C10—C6—P2	127.20 (12)	C44—C45—H45	120.1
C7—C6—Fe	69.92 (9)	C46—C45—H45	120.1
C10—C6—Fe	69.53 (9)	C45—C46—C41	120.03 (17)
P2—C6—Fe	125.62 (8)	C45—C46—H46	120.0
C8—C7—C6	107.86 (14)	C41—C46—H46	120.0
C8—C7—Fe	70.43 (9)		
			
C11—P1—C1—C5	−145.23 (13)	C1—P1—C11—C12	117.38 (13)
C21—P1—C1—C5	104.46 (14)	C21—P1—C11—C12	−130.95 (13)
S1—P1—C1—C5	−19.57 (15)	S1—P1—C11—C12	−7.64 (15)
C11—P1—C1—C2	35.24 (17)	C1—P1—C11—C16	−64.36 (14)
C21—P1—C1—C2	−75.08 (16)	C21—P1—C11—C16	47.30 (14)
S1—P1—C1—C2	160.89 (13)	S1—P1—C11—C16	170.61 (11)
C11—P1—C1—Fe	−57.11 (12)	C16—C11—C12—C13	0.0 (2)
C21—P1—C1—Fe	−167.42 (9)	P1—C11—C12—C13	178.28 (13)
S1—P1—C1—Fe	68.54 (11)	C11—C12—C13—C14	−0.3 (3)
C5—C1—C2—C3	0.08 (17)	C12—C13—C14—C15	0.9 (3)
P1—C1—C2—C3	179.67 (12)	C13—C14—C15—C16	−1.1 (3)
Fe—C1—C2—C3	−59.75 (11)	C14—C15—C16—C11	0.8 (2)
C5—C1—C2—Fe	59.82 (11)	C12—C11—C16—C15	−0.3 (2)
P1—C1—C2—Fe	−120.58 (14)	P1—C11—C16—C15	−178.54 (12)
C1—C2—C3—C4	−0.18 (18)	C1—P1—C21—C26	16.91 (16)
Fe—C2—C3—C4	−59.51 (11)	C11—P1—C21—C26	−95.38 (14)
C1—C2—C3—Fe	59.33 (11)	S1—P1—C21—C26	140.79 (12)
C2—C3—C4—C5	0.21 (18)	C1—P1—C21—C22	−163.87 (13)
Fe—C3—C4—C5	−58.72 (11)	C11—P1—C21—C22	83.84 (14)
C2—C3—C4—Fe	58.93 (11)	S1—P1—C21—C22	−39.99 (15)
C3—C4—C5—C1	−0.16 (18)	C26—C21—C22—C23	1.9 (3)
Fe—C4—C5—C1	−58.83 (11)	P1—C21—C22—C23	−177.31 (13)
C3—C4—C5—Fe	58.66 (11)	C21—C22—C23—C24	−1.3 (3)
C2—C1—C5—C4	0.05 (17)	C22—C23—C24—C25	−0.2 (3)
P1—C1—C5—C4	−179.57 (11)	C23—C24—C25—C26	1.2 (3)
Fe—C1—C5—C4	59.55 (11)	C22—C21—C26—C25	−1.0 (2)
C2—C1—C5—Fe	−59.50 (11)	P1—C21—C26—C25	178.21 (13)
P1—C1—C5—Fe	120.88 (12)	C24—C25—C26—C21	−0.6 (3)
C31—P2—C6—C7	91.76 (14)	C6—P2—C31—C36	−9.64 (15)
C41—P2—C6—C7	−157.72 (13)	C41—P2—C31—C36	−120.81 (14)
S2—P2—C6—C7	−32.20 (15)	S2—P2—C31—C36	116.17 (13)
C31—P2—C6—C10	−88.52 (15)	C6—P2—C31—C32	172.81 (13)
C41—P2—C6—C10	21.99 (16)	C41—P2—C31—C32	61.64 (15)
S2—P2—C6—C10	147.51 (12)	S2—P2—C31—C32	−61.38 (14)
C31—P2—C6—Fe	−178.74 (9)	C36—C31—C32—C33	0.9 (3)
C41—P2—C6—Fe	−68.22 (11)	P2—C31—C32—C33	178.53 (14)
S2—P2—C6—Fe	57.30 (11)	C31—C32—C33—C34	−1.4 (3)
C10—C6—C7—C8	0.13 (17)	C32—C33—C34—C35	0.2 (3)
P2—C6—C7—C8	179.89 (11)	C33—C34—C35—C36	1.4 (3)
Fe—C6—C7—C8	59.83 (11)	C32—C31—C36—C35	0.8 (2)
C10—C6—C7—Fe	−59.70 (11)	P2—C31—C36—C35	−176.76 (13)
P2—C6—C7—Fe	120.06 (12)	C34—C35—C36—C31	−1.9 (2)
C6—C7—C8—C9	−0.11 (18)	C6—P2—C41—C46	107.95 (14)
Fe—C7—C8—C9	58.68 (11)	C31—P2—C41—C46	−140.78 (13)
C6—C7—C8—Fe	−58.79 (10)	S2—P2—C41—C46	−18.60 (15)
C7—C8—C9—C10	0.06 (18)	C6—P2—C41—C42	−72.25 (14)
Fe—C8—C9—C10	58.34 (11)	C31—P2—C41—C42	39.01 (15)
C7—C8—C9—Fe	−58.28 (11)	S2—P2—C41—C42	161.20 (11)
C8—C9—C10—C6	0.02 (18)	C46—C41—C42—C43	−1.6 (2)
Fe—C9—C10—C6	59.08 (11)	P2—C41—C42—C43	178.63 (13)
C8—C9—C10—Fe	−59.05 (11)	C41—C42—C43—C44	0.6 (3)
C7—C6—C10—C9	−0.09 (17)	C42—C43—C44—C45	0.6 (3)
P2—C6—C10—C9	−179.85 (12)	C43—C44—C45—C46	−0.9 (3)
Fe—C6—C10—C9	−60.04 (11)	C44—C45—C46—C41	−0.1 (3)
C7—C6—C10—Fe	59.94 (10)	C42—C41—C46—C45	1.3 (3)
P2—C6—C10—Fe	−119.81 (13)	P2—C41—C46—C45	−178.86 (13)

### Hydrogen-bond geometry (Å, º)

Cg1 is the centroid of the C31–C36 benzene ring.

**Table:**

*D*—H···*A*	*D*—H	H···*A*	*D*···*A*	*D*—H···*A*
C2—H2···*Cg*1^i^	0.95	2.92	3.6111 (18)	130

Symmetry code: (i) *x*−1, *y*, *z*.
